# Pygopus2 inhibits the efficacy of paclitaxel-induced apoptosis and induces multidrug resistance in human glioma cells

**DOI:** 10.18632/oncotarget.15843

**Published:** 2017-03-02

**Authors:** Cefan Zhou, Hongxia Cheng, Wenying Qin, Yi Zhang, Hui Xiong, Jing Yang, Huang Huang, Yefu Wang, Xing-Zhen Chen, Jingfeng Tang

**Affiliations:** ^1^ Institute of Biomedical and Pharmaceutical Sciences, Key Laboratory of Fermentation Engineering (Ministry of Education), College of Bioengineering, Hubei University of Technology, Wuhan, 430068, China; ^2^ The State Key Laboratory of Virology, College of Life Sciences, Wuhan University, Wuhan, 430072, China; ^3^ Department of Chemical and Pharmaceutical Engineering, Wuhan Huaxia University of Technology, Wuhan, 430223, China; ^4^ XiLi People's Hospital, Shenzhen, Guangdong, 518055, China; ^5^ Institute for Immunology, Tsinghua University, Beijing, 100084, China; ^6^ Membrane Protein Disease Research Group, Department of Physiology, Faculty of Medicine and Dentistry, University of Alberta, Edmonton, AB, T6G 2R3, Canada

**Keywords:** Pygo2, paclitaxel, MDR1, P-glycoprotein, glioma

## Abstract

Anti-microtubule drugs, such as paclitaxel (PTX), are extensively used for the treatment of numerous cancers. However, growing evidence has shown that PTX resistance, either intrinsic or acquired, frequently occurs in patients and results in the failure of treatment, contributing to the high cancer mortality rate. Therefore, it is necessary to identify the genes or pathways involved in anti-microtubule drug resistance for future successful treatment of cancers. Pygopus2 (Pygo2), which contains a Zn-coordinated plant homeodomain (PHD) finger domain, is critical for β-catenin-dependent transcriptional switches in normal and malignant tissues and is over-expressed in various cancers, including human brain glioma. In this study, we report that over-expression of Pygo2 inhibited the efficacy of PTX and contributed to cell multidrug resistance in two different ways. First, over-expression of Pygo2 inhibited the PTX-induced phosphorylation of B-cell lymphoma 2 (Bcl-2), suppressing the proteolytic cleavage of procaspase-8/9 and further inhibiting the activation of caspase-3, which also inhibits the activation of the JNK/SAPK pathway, ultimately inhibiting cell apoptosis. Second, over-expression of Pygo2 facilitated the expression of P-glycoprotein, which acts as a drug efflux pump, by promoting the transcription of Multi-drug resistance 1 (MDR1) at the MDR1 promoter loci, resulting in acceleration of the efflux of PTX.

## INTRODUCTION

Paclitaxel (PTX) is one of the most effective anticancer agents available clinically, and it has a wide spectrum of activity against solid tumors [1]. It functions as an inhibitor of the dynamic instability of microtubules, which is crucial during mitosis because the polymerization dynamics of the mitotic spindle accomplishes the proper alignment and segregation of the chromosomes to form daughter cells [2]. Preventing the requisite dynamics leads to arrest of mitosis at G2/M phase, loss of mitochondrial transmembrane potential, phosphorylation of Bcl-2, activation of caspases and ultimately apoptosis [3]. Many types of solid tumors, including human brain glioma, are treated with PTX alone or in combination with other chemotherapeutic agents. Previous studies have demonstrated that PTX can be delivered by mesenchymal stem cells (MSCs) and kill glioblastoma cells [4, 5] when MSCs loaded with PTX are in close proximity [6]. In addition, conjugated linoleic acid-paclitaxel (CLA-PTX), which can access the brain tissue and target brain tumors, was synthesized and showed great potential to become a new prodrug of PTX [7]. However, despite being one of the most widely used chemotherapeutics for solid tumors, the exact mechanisms and the factors that govern the anticancer function of PTX are not completely understood [8]. Growing evidence has shown that PTX resistance has become more frequent, and it is known that drug resistance is a multifactorial process that likely develops through a series of genetic and/or protein modifications. However, the mechanism responsible for chemo-resistance in glioma remains poorly understood [9–11]. Resistance to PTX has been confirmed to be associated with over-expression of P-glycoprotein (P-gp) [10], encoded by the multidrug resistance gene *MDR1*, which functions as a drug efflux pump [12, 13]. The transcription of *MDR1* can be regulated by several pathways due to the complex pattern of the promoter [14]. Moreover, epigenetic regulation, including histone modification and DNA methylation, adds more complexity and has been extensively studied over the past several years [15, 16].

Dysregulation of the canonical Wnt/β-catenin signaling pathway is associated with the development and progression of many malignancies. A growing body of evidence indicates that Pygopus2 (Pygo2) acts as an important coactivator of the Wnt/β-catenin transcriptional complex, and functions as an enhancer of β-catenin activity in the classical Wnt pathway [17]. There is a highly conserved structure called the PHD domain with a Zn^2+^ coordinating finger at the C terminus of Pygo2 [18]; previous studies have shown that PHD-containing proteins can act as protein code readers to link the chromatin remodeling complex to specific changes in gene transcription, as demonstrated for Wnt/β-catenin target genes. For example, Pygo2 was found to possess the ability to directly bind to histone H3 trimethylated at lysine 4 (H3K4me3) [19], associate with histone-modifying enzymes and recruits them to recruit chromatin to facilitate H3K4 trimethylation and histone acetylation in breast cancer cells [20]. Pygo2 has been reported to be over-expressed in several malignant tumors, including those of epithelial ovarian cancer, breast cancer and human glioma, and appears to play an important role in the growth of these tumors. [21–23]. Moreover, our previous study found that Pygo2 mRNA and protein expression were significantly higher in glioma tissues and cells, and abnormally high expression of Pygo2 protein in glioma patients was correlated with a poor prognosis. In addition, knockdown of Pygo2 inhibits glioma cell proliferation, migration and invasion [24]. However, the relationship between Pygo2 and multidrug resistance has been rarely studied. The present work aims to investigate the molecular mechanism of Pygo2-mediated inhibition of the efficacy of PTX-induced apoptosis and its role in multidrug resistance.

## RESULTS

### Pygo2 promotes the proliferation of glioma cells treated with PTX

**Figure 1:**
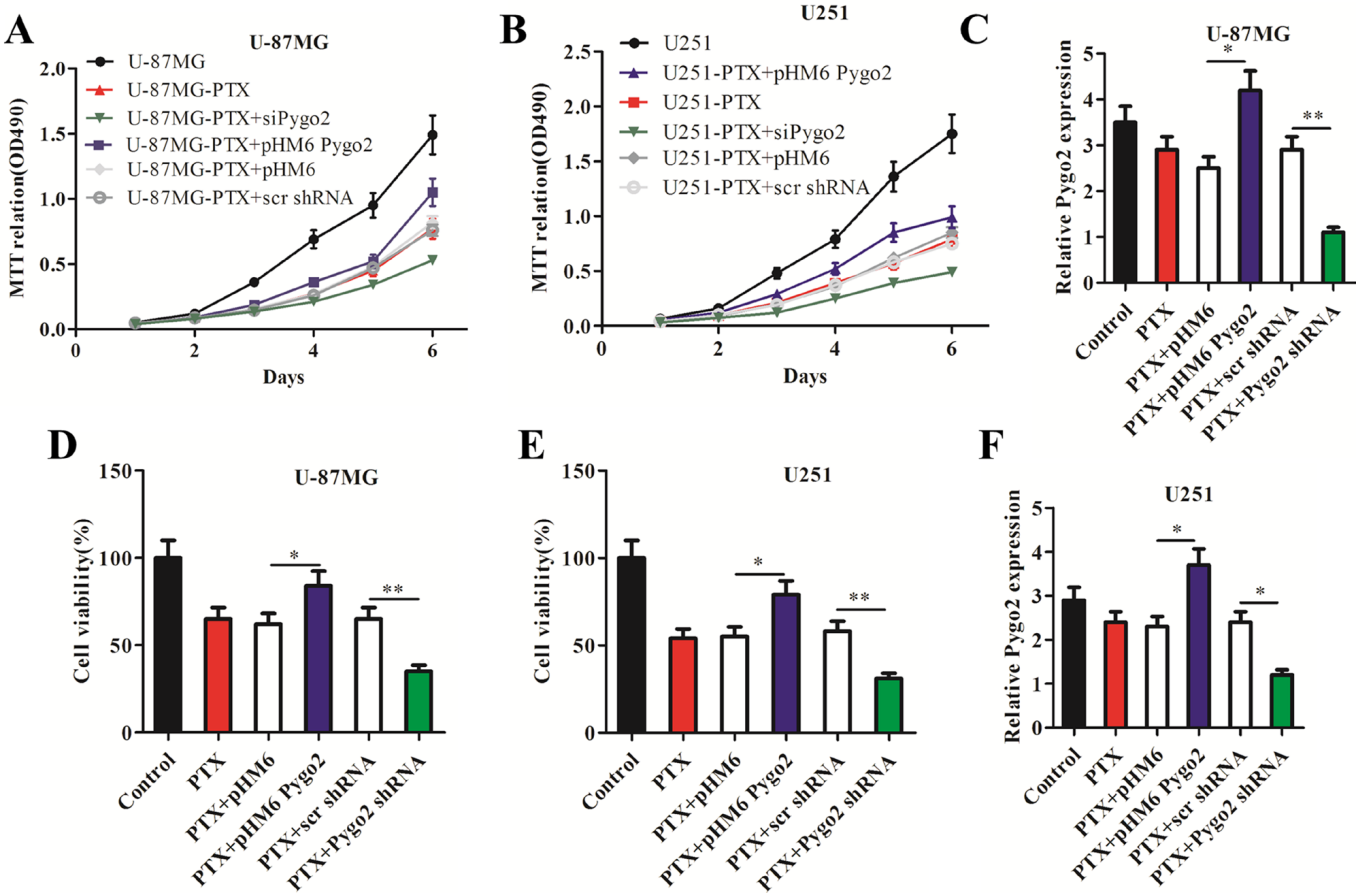
Pygo2 promotes the proliferation of glioma cells treated with paclitaxel Six groups of cells (PTX, PTX+ pHM6-Pygo2, PTX+Pygo2 shRNA, PTX+pHM6, PTX+scr shRNA) were seeded at a density of 1×10^5^ cells/well in 6-well plates and treated with 30 nM PTX for 48 h; normal cells were used as a control. (**A**–**B**) An MTT assay revealed that Pygo2 counteracted the effect of PTX-induced cell growth arrest in human glioma U-87MG or U251 cell lines. (**C**, **F**) qRT-PCR showed the relative Pygo2 mRNA expression level in the treated cell lines. Pygo2 expression was normalized to β-actin. (**D**–**E**) Pygo2 rescued the cell viability of U-87MG and U251 cells. Data are presented as the mean ± SD of three independent experiments. Two-tailed Student's *t*-test was used; *P < 0.05 and **P < 0.01.

PTX is commonly used as an anti-tumor drug that blocks mitosis and kills tumor cells by suppressing microtubule dynamics. We previously reported that Pygo2 was up-regulated in human brain glioma tissues and cell lines [24]. To investigate the effect of Pygo2 expression on PTX-induced cell growth arrest in human brain glioma U-87MG and U251 cell lines, several groups of stably transfected cell lines were constructed by transfecting cells with recombinant expression plasmids encoding pHM6, pHM6-Pygo2, pHM6-Pygo2 shRNA and pHM6-scr shRNA. The relative Pygo2 mRNA expression was detected by qRT-PCR in human glioma U-87MG (Figure 1C) and U251 (Figure 1F) cell lines after treatment with 30 nM PTX for 48 h. MTT and trypan blue dye exclusion assays were performed to examine the effects of exogenous Pygo2 on the proliferation and viability, respectively, of the human glioma cell line U-87MG (Figure 1A and 1D). As expected, the viability of cells treated with PTX was more depressed than that of untreated cells (control). However, over-expression of Pygo2 (PTX+pHM6 Pygo2) promoted cell viability and resulted in an increased cell proliferation rate in a time-dependent manner. In contrast, silencing of Pygo2 enhanced the anti-proliferative effect of PTX. Consistent results were observed in U251 cells (Figure 1B and 1E). Overall, our results indicate that Pygo2 enhances the proliferation of glioma cell lines treated with PTX and promotes PTX multidrug resistance in human brain glioma U-87MG and U251 cells.

### Pygo2 promotes metastasis in glioma cells treated with PTX

**Figure 2:**
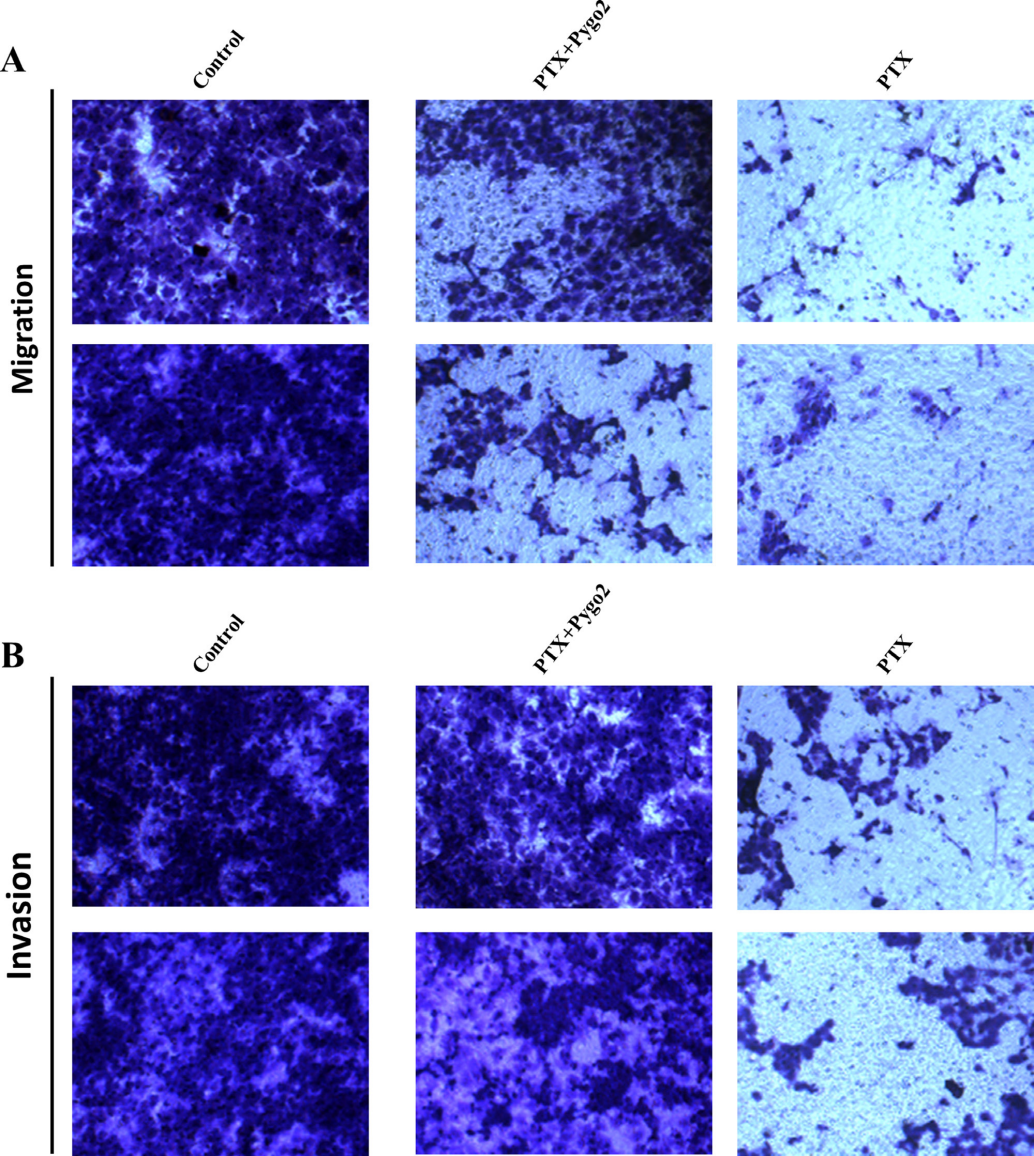
Effect of Pygo2 on cell metastasis of U-87MG cells treated with PTX Transwell assays show the effects of Pygo2 on U-87MG cell migration and invasion. Representative micrographs exhibiting the effects of Pygo2 on cell migration (**A**) and invasion (**B**).

Cell migration and invasion were further evaluated in glioma U-87MG cells treated with PTX by examining the effects of exogenous Pygo2. Transwell assays were used to demonstrate the effect of Pygo2 on cell migration. Compared with PTX-treated cells, cells transfected with exogenous Pygo2 exhibited a significantly increased migratory ability (Figure 2A). To examine the effect of Pygo2 on cell invasion, U-87MG cells were cultured in Transwell chambers pre-coated with matrigel for 8 h prior to measurements. We found that increased Pygo2 expression significantly increased the ability of the cells to cross the matrigel-coated inserts (Figure 2B). Taken together, our results demonstrated that high Pygo2 levels enhance the invasion and migration capacity of human glioma U-87MG cells treated with PTX.

### Pygo2 inhibits PTX-induced apoptosis in glioma cells

**Figure 3:**
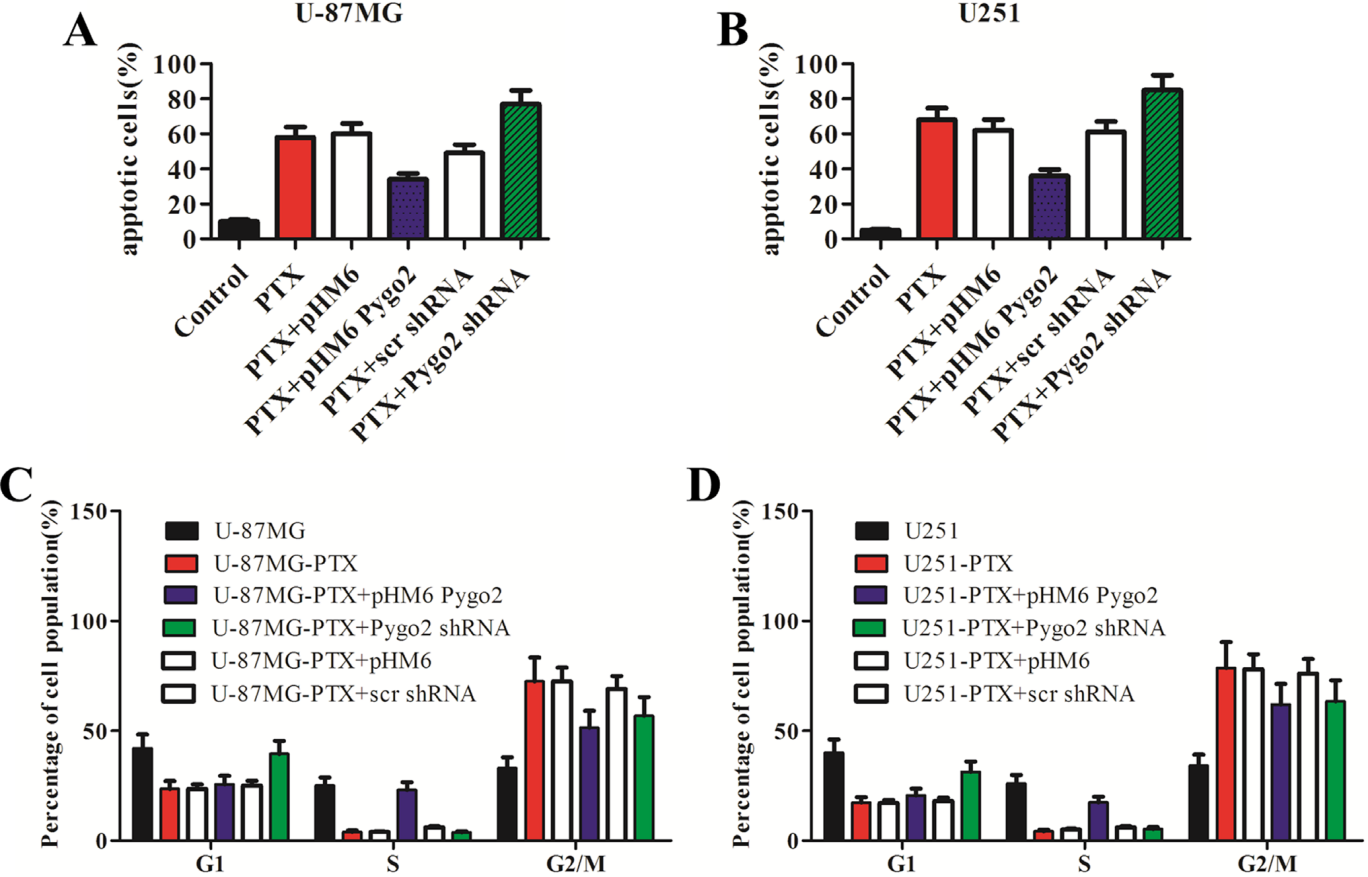
Pygo2 inhibits the PTX-induced apoptosis and G2/M arrest of glioma cells Cells were treated with 30 nM PTX for 48 h, and the percentage of apoptotic cells (**A**–**B**) and mitotic cells (**C**–**D**) was determined by flow cytometry after staining the cells with annexin V-FITC and PI. Decreased apoptosis rates were found in the cells expressing exogenous Pygo2 protein after treatment with PTX compared to the cells with low levels of Pygo2, and the sub-G1 index was increased in the Pygo2-knockdown cells. Data are presented as the mean ± SD of three independent experiments.

It is generally accepted that anti-microtubule agents such as vincristine and PTX can induce apoptosis [25, 26]. To further understand how enforced expression of Pygo2 inhibits the efficacy of PTX in inducing cell proliferation arrest, we evaluated the apoptosis rate in the six different stably transfected cell lines using flow cytometry after staining the cells with annexin V-FITC and propidium iodide (PI). We found that the percentage of apoptotic cells increased to 58.2% after treatment with PTX, and cells transfected with pHM6 and pHM6-scr shRNA vector were not significantly different. We noticed that the percentage of apoptotic cells was much lower in the cells expressing exogenous Pygo2 protein (PTX+pHM6-Pygo2) compared with cells treated with PTX alone. In contrast, silencing of Pygo2 (PTX+Pygo2 shRNA) enhanced PTX-induced apoptosis (Figure 3A–3B).

### Pygo2 inhibits PTX-induced cell cycle arrest

Cell cycle profiles of PTX-treated human glioma cells were evaluated using flow cytometry. A clear G2/M arrest was observed in the cells that were stably transfected with either pHM6 or pHM6-scr shRNA vector. A decreased percentage of G2/M cells were found in the cells expressing exogenous Pygo2 protein. In contrast, in the cells expressing low levels of Pygo2 there was also a decreased percentage of G2/M cells found, but this decrease was accompanied by an increase in the percentage of sub-G1 phase cells (Figure 3C–3D). The result in U-87MG and U251 cells was similar. These results indicate that Pygo2 silencing enhances the drug-induced apoptosis partly via accumulation of cells in subG1 phase.

### Pygo2 inhibits the activation of the JNK/SAPK pathway

**Figure 4:**
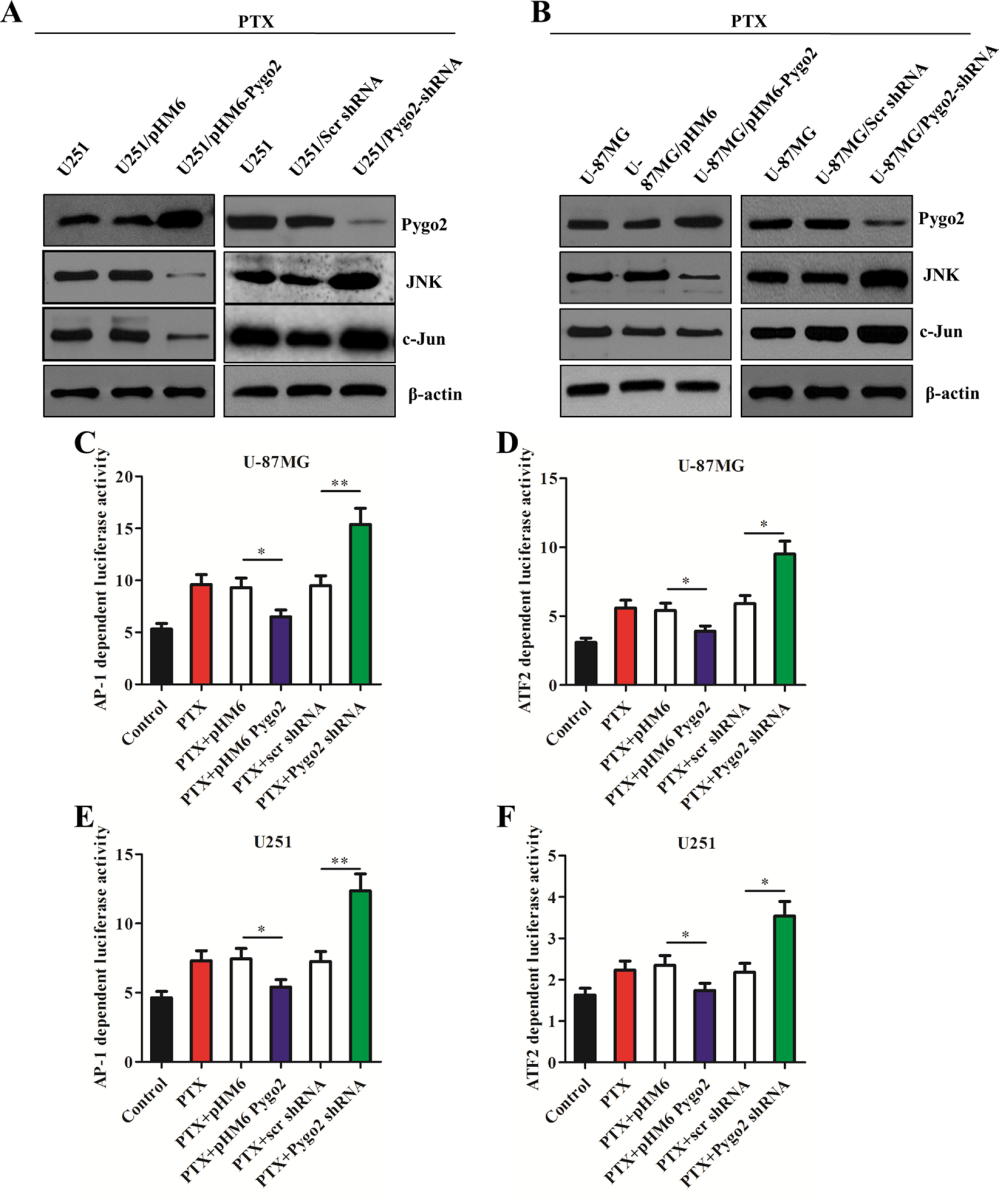
Pygo2 inhibits the activation of the JNK/SAPK pathway and the AP-1 and ATF2 activity (**A**–**B**) The protein levels in the stably transfected cell lines were detected by western blot analysis after treatment with 30 nM PTX for 48 h; β-actin was used as a control. (**C**, **E**) AP-1 transcription activity was examined using the reporter plasmid pAP-1-Luc. (**D**, **F**) ATF2 transcription activity was examined using the fusion trans-activator plasmid pFA-ATF2. Data are presented as the mean ± SD of three independent experiments. *P < 0.05 and **P < 0.01.

It is well known that the JNK/SAPK cascade has an apoptosis-promoting role, specifically in apoptotic processes resulting from microtubular dysfunction [27, 28], and activation of the JNK/SAPK pathway can be induced by PTX [29]. We also detected activation of the JNK/SAPK pathway following treatment with PTX (Figure 4A–4B). To investigate the relationship between the expression level of Pygo2 and the contribution of the JNK/SAPK pathway to PTX-induced apoptosis, we first examined the JNK protein levels in the stably transfected cell lines treated with PTX. Western blot analysis demonstrated that the level of JNK was significantly reduced in the cells expressing exogenous Pygo2 protein compared with normal U251 or U-87MG cells. In contrast, there was an increased level of JNK in the cells expressing low levels of Pygo2 (Figure 4A–4B). The results of a western blot analysis of c-Jun, a substrate of JNK, were similar to those of JNK.

We also tested the effect of Pygo2 on the transcriptional activity of AP-1, which is regulated by the JNK/SAPK signaling pathway, using an AP-1 reporter plasmid (pAP-1-luci) (Figure 4C, 4E). Decreased activity was detected in the cells expressing exogenous Pygo2. The results of experiments with the transcription factor ATF2, which is phosphorylated and activated by JNK, confirmed this conclusion. Together, these data demonstrate that Pygo2 inhibits the apoptotic efficiency of PTX by modulating the JNK/SAPK signaling pathway and inducing a decrease in AP-1 activity.

### Pygo2 inhibits the phosphorylation of Bcl-2 and the activation of caspases

**Figure 5:**
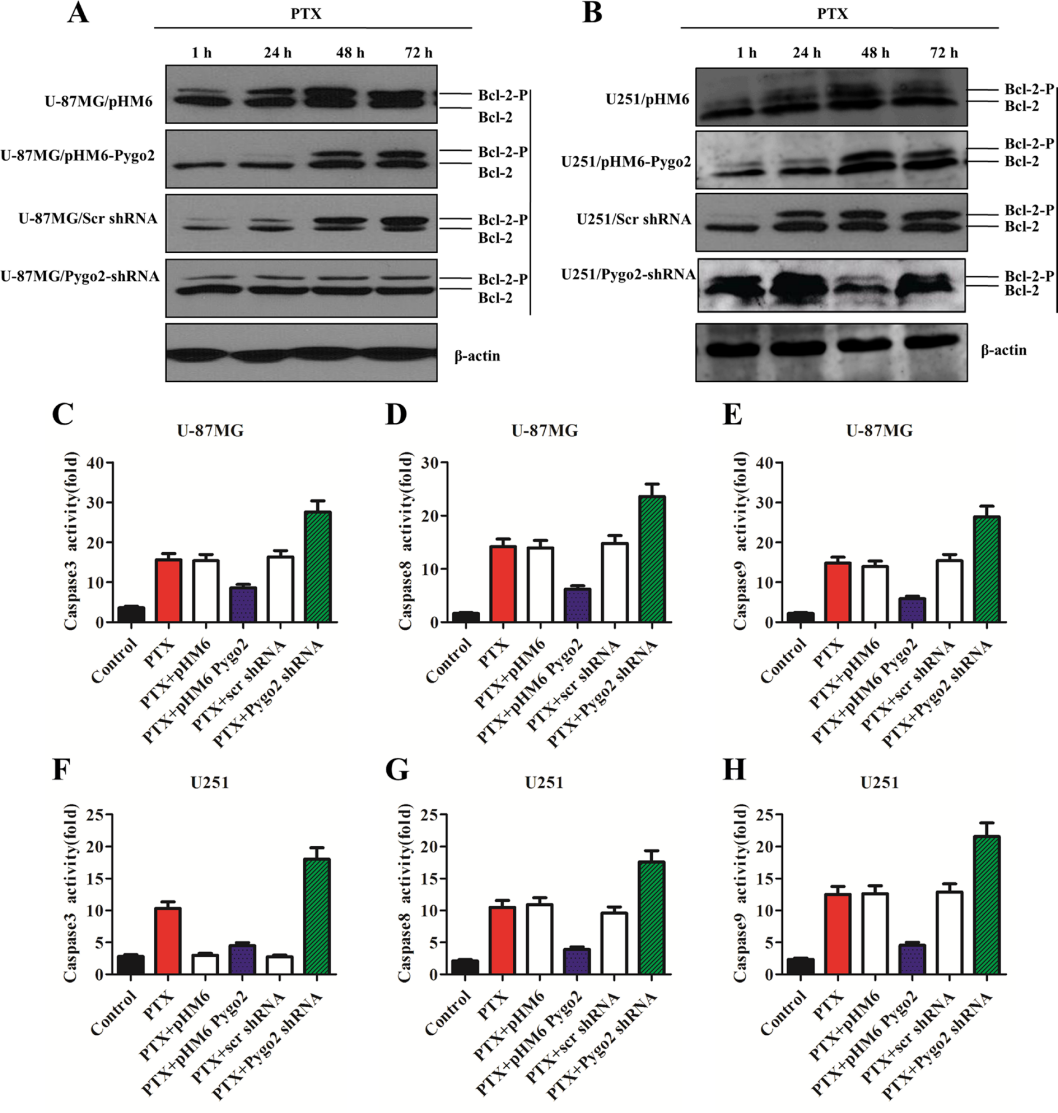
Pygo2 inhibits the phosphorylation of Bcl-2 and the activation of caspases (**A**–**B**) The protein levels in the stably transfected cell lines were detected by western blot analysis after treatment with 30 nM PTX in a time-dependent manner; β-actin was used as a control. (**C**–**F**) caspase-3/7 activities were measured with a Caspase-Glo^®^ 3/7 Assay kit. (**D**–**G**) Caspase-8 activity was measured with a Caspase-Glo^®^ 8 Assay kit. (**E**–**H**) Caspase-9 activity was measured with a Caspase-Glo^®^ 9 Assay kit. Data are presented as the mean ± SD of three independent experiments.

Because the phosphorylation of Bcl-2 induced by PTX also plays a critical role in apoptotic decisions [30], we also studied the relationship between the Pygo2 expression level and the phosphorylation level of Bcl-2 in human brain glioma U-87MG and U251 cell lines. To investigate the effect of Pygo2 on PTX-induced phosphorylation of Bcl-2, the stably transfected cell lines were treated with 30 nM PTX in a time-dependent manner. Bcl-2 phosphorylation was examined by western blot. As shown in Figure 5A–5B, immunoblotting with anti-Bcl-2 antibody revealed two bands of 30 and 28 kDa in extracts from the control cells at the 24 h and 48 h time points. However, the 30 kDa band disappeared 72 h later. We discovered the same PTX-induced mobility shift of the 30 kDa band, but at a later time point, with over-expression of exogenous Pygo2. In addition, the mobility shift of the 30 kDa band appeared much earlier when endogenous Pygo2 was knocked down. This indicates that PTX induced Bcl-2 phosphorylation, as evidenced by the presence of several immunoreactive bands, was inhibited by over-expression of exogenous Pygo2. In contrast, the phosphorylation of Bcl-2 induced by PTX was more sensitive when endogenous Pygo2 was knocked down.

To investigate the effects of Pygo2 on PTX-induced activation of caspase proteins and other apoptosis-related proteins, Caspase-Glo^®^ 3/7, Caspase-Glo^®^ 8 and Caspase-Glo^®^ 9 assays were performed. The stably transfected cell lines were treated with 30 nM PTX for 48 h, and then, a proluminescent caspase substrate and a proprietary thermostable luciferase was added. The substrate underwent cleavage and generated a “glow-type” luminescent signal that was proportional to the activities of caspase-3/8/9. We found that the cells that over-expressed exogenous Pygo2 had less caspase-3, −8 and −9 activity compared to the control cells. In contrast, low Pygo2 expression showed more effect on the activation of caspase-3, −8 and −9 (Figure 5C–5H). Together, these data demonstrate that Pygo2 inhibits the apoptotic efficiency of PTX, in part by modulating Bcl-2 phosphorylation and reducing the activation of caspases.

### Pygo2 up-regulates the expression of P-gp

**Figure 6:**
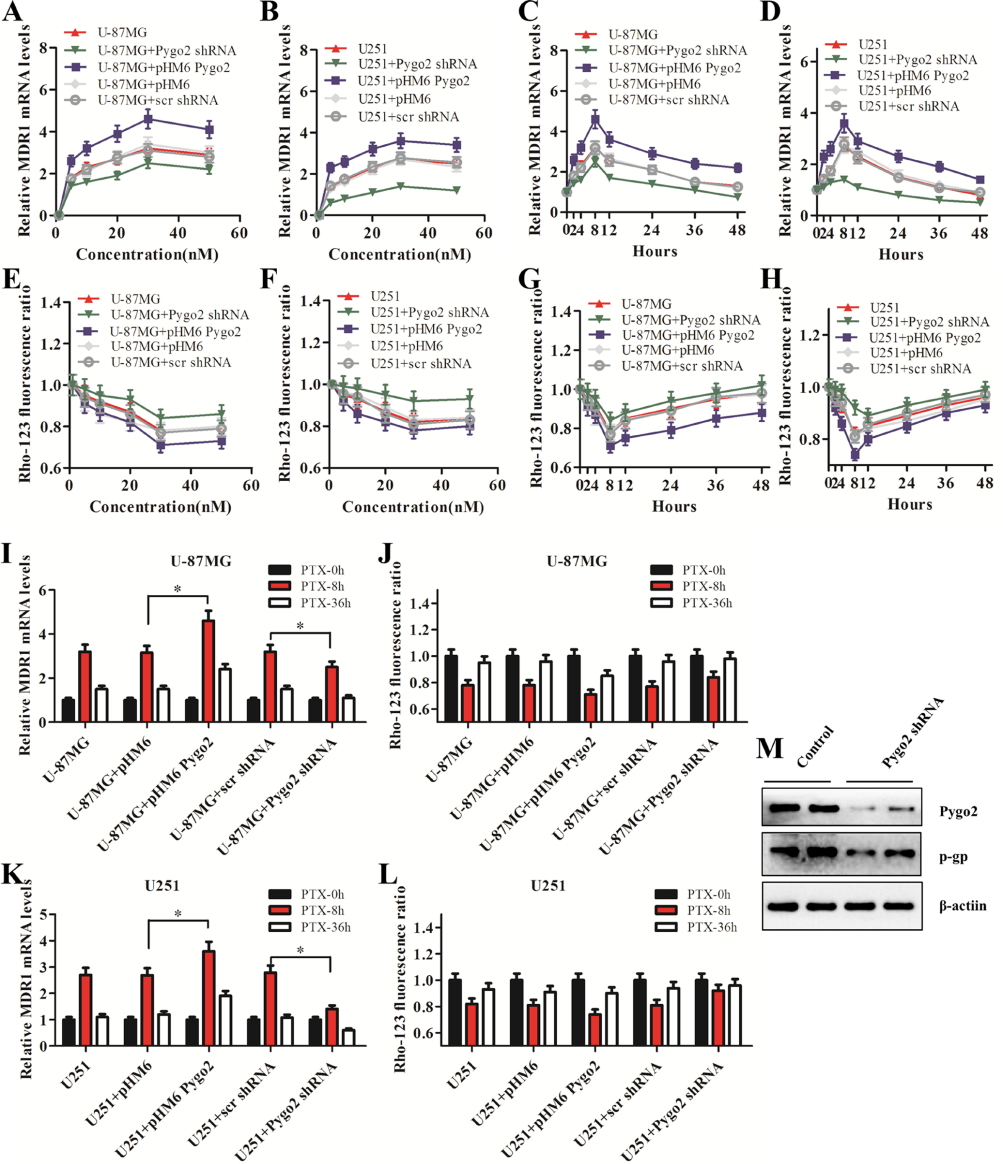
Pygo2 up-regulates the expression of P-glycoprotein (**A**–**B**) The effect of Pygo2 on the mRNA levels of *MDR1* in a PTX concentration-dependent manner. The cells were treated with PTX for 8 h. (**C**–**D**) The effect of Pygo2 on the mRNA levels of *MDR1* in a PTX-treatment-time-dependent manner. The cells were treated with 30 nM PTX. (**E**–**H**) Rho-123 fluorescence ratio in the stably transfected U-87MG and U251 cells after PTX treatments at different concentrations and time points. (**I**, **K**) *MDR1* mRNA levels in U-87MG and U251 cells after 8 h/36 h of PTX treatment. (**J**, **L**) Rho-123 fluorescence ratio in U-87MG and U251 cells after 8 h/36 h of PTX treatment. (**M**) P-gp was down-regulated after Pygo2 was knocked down in PTX-treated cells. *MDR1* expression was normalized to β-actin. Data are presented as the mean ± SD of three independent experiments. *P < 0.05 and **P < 0.01.

P-gp, encoded by the *MDR1* gene, is the most extensively studied human ATP-binding cassette (ABC) transporter. We found that the expression of *MDR1* was reduced after endogenous Pygo2 was knocked down (Figure 6M). To further investigate the regulatory relationship between Pygo2 and *MDR1* expression and how human brain glioma cells may develop multidrug resistance to PTX through *MDR1*, the stably transfected U-87MG and U251 cells were induced by PTX in a time course experiment, and the *MDR1* expression was evaluated. The selected concentrations of PTX were 1, 5, 10, 20, 30 and 50 nM. A concentration of 30 nM was chosen for the next experiment because it had the highest *MDR1*-inducing effect (Figure 6A–6B). It was revealed that P-gp was induced after treatment with PTX, and the induction reached a maximum at the 8 h time point (Figure 6C–6D) and was down-regulated following a more prolonged exposure time, indicating that P-gp induction was an early event. In addition, we discovered that the *MDR1* expression level was much higher in the cells expressing exogenous Pygo2 compared with control cells, and in contrast, there was a decreased level of *MDR1* in the cells expressing low levels of Pygo2.

We further investigated P-gp transport activity using the fluorescent substrate Rho-123. The activity of P-gp was measured by examining the intracellular accumulation of Rho-123 [31]. The activity of P-gp also reached a maximum at the same concentration and time points determined in the qRT-PCR assay (Figure 6E–6L). Together, these data demonstrate that Pygo2 inhibits the apoptotic efficiency of PTX, in part by facilitating the expression of P-gp, which functions as a drug efflux pump that pumps intracellular PTX out of the cell.

### Pygo2 functions as a transcriptional co-regulator of P-gp

**Figure 7:**
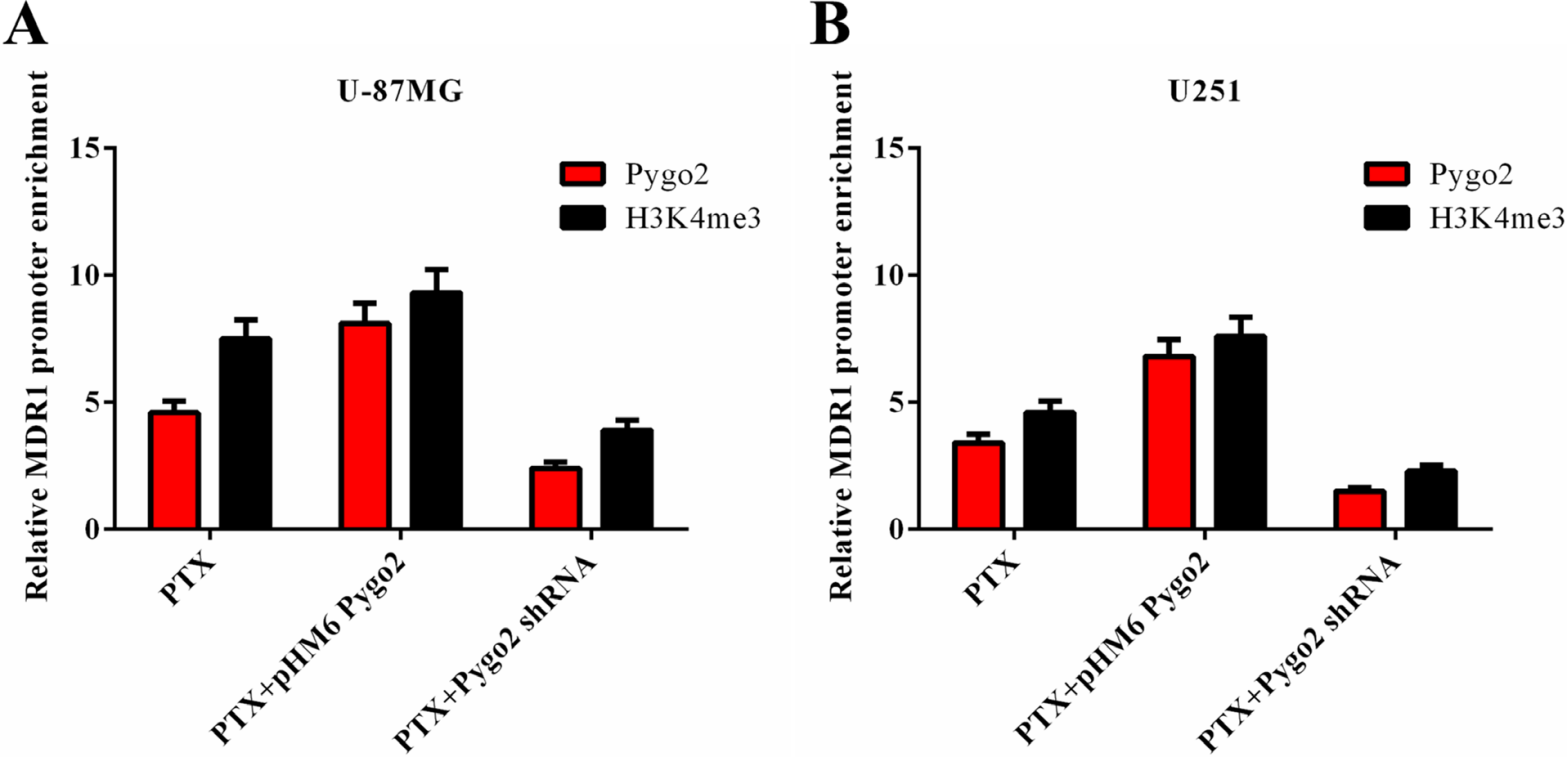
Effect of Pygo2 on the recruitment of transcriptional regulators to the *MDR1* promoter Pygo2 and H3K4me3 occupied the *MDR1* promoter following treatment with 30 nM PTX for 8 h in human brain glioma U-87MG (**A**) and U251 (**B**) cell lines.

Previous studies have indicated that the PHD domain can act as a transcriptional co-activator in the presence of RNA polymerase II (RNAPII), and this activity is dependent on binding of the PHD finger to H3K4me3 [32, 33]. To investigate the possible molecular mechanism of Pygo2 up-regulation of the expression of P-gp, a CHIP assay was performed using specific polyclonal antibodies against Pygo2 and H3K4me3. Binding of the *MDR1* DNA promoter-specific fragment was detected. We found that the two transcriptional factors were both present on *MDR1* promoter loci, and the relative *MDR1* mRNA promoter enrichment was reduced after endogenous Pygo2 was knocked down, accompanied by the down-regulation of H3K4me3. In contrast, there were increased levels of H3K4me3 on the *MDR1* promoter loci after exogenous Pygo2 expression (Figure 7A–7B). These data indicated that Pygo2 functions as a transcriptional co-regulator when *MDR1* expression is induced by treatment with PTX.

## DISCUSSION

PTX, which is used to treat human brain glioma, acts via a distinct mechanism [34]. It binds to tubulin, promoting microtubule assembly and stabilizing microtubules by bundle formation. However, the efficacy of PTX-based chemotherapy for cancer treatment has been impeded by drug resistance [35]. The mechanisms of acquired resistance have been extensively studied by generating cell models in the laboratory and examining cellular processes such as decreased drug uptake into cells, activation of detoxifying enzymes, increased drug efflux and inhibition of apoptotic signaling pathways [36]. In this work, we aimed to determine the role of Pygo2 in the drug resistance of human glioma cells after treatment with PTX. As a result, we found that Pygo2 inhibits the efficacy of PTX-induced apoptosis. The molecular mechanism may involve accumulation of cells in sub-G1 phase, modulation of the JNK/SAPK signaling pathway, decreased AP-1 and ATF2 activity, modulation of Bcl-2 phosphorylation, reduced caspase activity and facilitation of the expression of P-gp, which pumps intracellular PTX out of the cell.

According to the results of cell proliferation and cell viability assays, exposure of human U-87MG and U251 cells to moderate cytotoxic concentrations of PTX can induce cell growth arrest and cell apoptosis (Figure 1), and we noticed that silencing Pygo2 decreased the proliferation ratio of cells; it also enhanced the apoptotic efficiency of PTX in U-87MG and U251 cells. In contrast, over-expression of exogenous Pygo2 promoted cell proliferation and inhibited the PTX-induced apoptotic efficiency (Figure 2). Because the Bcl-2 family plays a critical decision-making role in cell survival and apoptosis, especially the subfamilies of the Bcl-2 protein, which have been shown to play important roles in the apoptotic response [37]. Bcl-2, specifically, was found to be phosphorylated after treatment with PTX [30]. Therefore, we set out to examine the relationship between the Pygo2 expression level and the phosphorylation of Bcl-2. Specifically, we found that the phosphorylation of Bcl-2 induced by PTX was inhibited by over-expression of exogenous Pygo2. In contrast, the band of phosphorylated Bcl-2 appeared earlier when Pygo2 was knocked down (Figure 4). In addition, a decreased percentage of G2/M cells was found in both Pygo2 over-expressing and Pygo2-silenced cells, but an increase in the percentage of sub-G1 phase cells was observed in the Pygo2-silenced cells (Figure 2), which was similar to the results of an earlier study [38].

We further examined how Pygo2 knockdown or Pygo2 expression impacted the activation of the JNK pathway. A low expression level of JNK was detected in cells over-expressing Pygo2 (Figure 3). The anti-apoptotic Bcl-2 proteins have been reported to be phosphorylated and inactivated by JNK, which is induced by microtubule-interacting agents, including PTX [39–41], although this conclusion is controversial because some studies indicate that phosphorylation may enhance the anti-apoptotic activity of Bcl-2 [42] and may stabilize Bcl-2 by preventing its ubiquitin-mediated degradation, which increases prosurvival signaling [43]. An earlier study also discovered that PTX-induced phosphorylation of Bcl-2 can occur in the absence of JNK activation [44]. Therefore, these data indicate that the JNK pathway may not be the physiologically relevant kinase pathway that induces Bcl-2 phosphorylation *in vivo*. Consistent with this hypothesis, the results of our study indicated that Pygo2 may function as an intermediary that regulates another kinase or a phosphatase that induces increased Bcl-2 phosphorylation, also possibility play a crucial role in regulating the activation of JNK though the upstream element. However, further investigation is necessary to delineate the mechanistic route of the apoptosis pathway induced by PTX or other anti-microtubule agents.

Apoptosis is initiated through two fundamental pathways: the extrinsic (death receptor-mediated) pathway, which is initiated through the activation of caspase-8 by death receptors, followed by the caspase-8-induced activation of an effector caspase (caspase-3); and the intrinsic (mitochondrial-mediated) pathway, which is initiated by the activation of caspase-9 following cytochrome c release from the mitochondria, which in turn activates caspase-3. As shown in Figure 7, we also revealed that silencing Pygo2 significantly enhances the activation of caspases 9, 8 and 3 in human U-87MG and U251 cells. Therefore, it seems that Pygo2 not only inhibits PTX-induced apoptosis through the extrinsic pathway but also through the intrinsic pathway. It is most likely that Pygo2 inhibits the disintegration of mitochondria induced by PTX, resulting in cytochrome *c* escape to the cytoplasm. Then, cytochrome *c* interacts with procaspase-9, leading to the activation of procaspase-9 through proteolytic cleavage [45, 46]. Cleaved caspase-9 in turn cleaves and activates procaspase-3 [47], with the effect of increasing the activation of the JNK pathway. The human glioma cells were led to apoptosis by cleaved caspase-3, which finally executed apoptosis by cleaving a number of protein substrates that are essential for DNA repair [47].

Growing evidence suggests that JNK is closely related to the expression of *MDR* [48, 49]. Here, using a CHIP assay, we demonstrated that Pygo2 was preloaded at the *MDR1* promoter before stimulation. It is possible that the JNK pathway regulates the expression of Pygo2 in a peculiar manner, which was not been revealed so far. Combined with the above results, Pygo2 protein appears to provide regulatory feedback to the JNK pathway through the phosphorylation of JNK, but this hypothesis requires further validation. In addition, we noticed that the expression of *MDR1* was increased in the cells that had a normal Pygo2 expression level following exposure to PTX. This indicates that there are other transcription activation factors that can regulate the expression of *MDR1*. We discovered that c-Jun was reduced when Pygo2 was highly expressed. c-Jun is a protein that forms the activator protein 1 (AP-1) early response transcription factor. It is well known that c-Jun can be activated through double phosphorylation on serine 63 and 73 that is mediated by the JNK pathway [50]. The data presented here suggest that c-Jun may play an important role in up-regulation of *MDR1* and P-gp expression.

**Figure 8:**
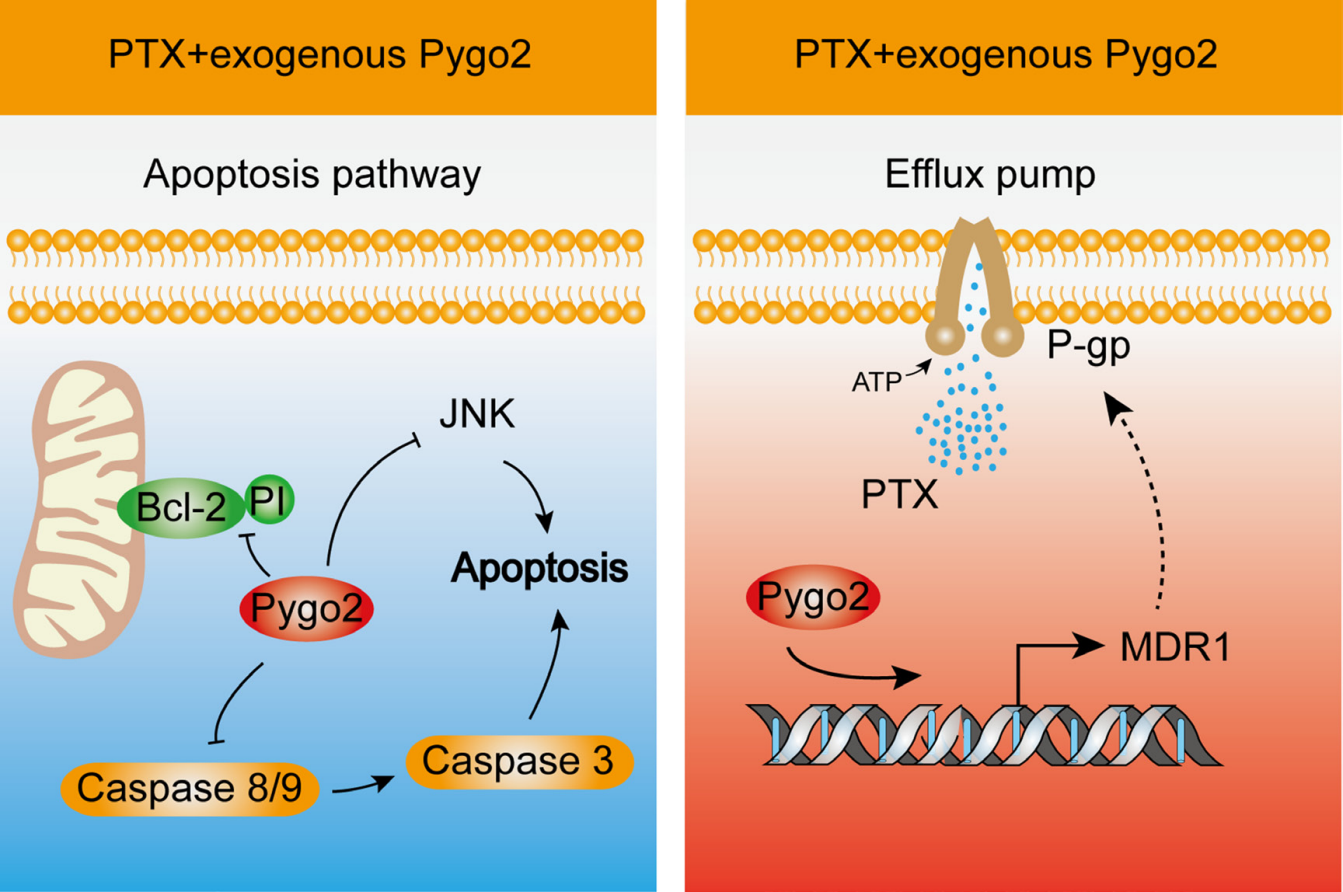
Schematic illustration showing how Pygo2 inhibits the efficacy of PTX-induced apoptosis in human glioma cells PTX treatment of cells causes a series of apoptotic responses, including phosphorylation of Bcl-2, activation of caspase-8/9, and activation of the JNK pathway, which were inhibited by over-expression of exogenous Pygo2 (Left). Cells also exhibited an increased expression level of *MDR1*. P-gp acts as an efflux pump that pumps the PTX granules out of cells, and up-regulated expression of P-gp further mediates PTX resistance. Over-expression of exogenous Pygo2, which occupies the promoter of *MDR1*, enhances the expression of P-gp to strengthen the multidrug resistance (Right).

In addition, the occupancy of H3K4me3 at the promoter was moderately increased in cells expressing exogenous Pygo2, and the transcription product of P-gp was clearly increased (Figure 8). In contrast, the enrichment of H3K4me3 was reduced when endogenous Pygo2 was silenced, similar to the results of an earlier study [51], which demonstrated that Pygo2 promotes Wnt target gene transcription by recruiting the mediator complex via interactions with Med12 and Med13 through the N-terminal domain of Pygo2. Pygo2 is generally considered to be an activating transcription factor of the Wnt pathway, but in a previous study, we also proposed that it plays an important role in activating the transcription of another gene. Here, we verified this assumption that Pygo2 could activate the transcription of other genes in addition to Wnt pathway target genes.

In conclusion, these results demonstrated that the molecular mechanism by which Pygo2 inhibits the efficacy of PTX-induced apoptosis in human glioma U-87MG and U251 cells involves two relevant activities. In the cytoplasm, over-expression of exogenous Pygo2 inhibited PTX-induced activation of the apoptosis pathway components. In the nucleus, elevated Pygo2 expression functioned as a transcription activation factor that promoted the expression of *MDR1*, facilitating the expression of P-gp, which pumps PTX granules out of the cells.

## MATERIALS AND METHODS

### Cell culture and transfection

The human brain glioma U-87MG and U251 cell lines were purchased from the Cell Center of the Institute of Biochemistry and Cell Biology, Chinese Academy of Sciences (Shanghai, China). Cells were cultured in Dulbecco's modified Eagle's medium (DMEM) (Gibco, USA) supplemented with 10% fetal bovine serum (Gibco, USA), 100 U/ml penicillin G and 100 μg/ml streptomycin at 37°C in a humidified incubator containing 5% CO_2_. Lipofectamine 2000 Transfection Reagent (Invitrogen, USA) was used to transfect the U251 and U-87MG cell lines according to the manufacturer's instructions. U251 and U-87MG cells were treated with Lipofectamine 2000 followed by transfection with recombinant pHM6-Pygo2 and pHM6-Pygo2 shRNA expression plasmids and scr-shRNA; pHM6 vector plasmid was used as a negative control.

### Survival (trypan blue dye exclusion) assay

Cells (1 × 10^5^ cells/well) were seeded into 6-well plates, treated with 30 nM PTX and incubated at 37°C in humidified atmosphere of 5% CO_2_ for 48 h. Cells were counted after staining with 0.4% (w/v) trypan blue dye using a dual-chamber counting slide under a light microscope. Non-viable cells were observed as blue-stained, while viable cells excluded the stain.

### Cell apoptosis and cycle assays

Cells were collected after treatment with PTX for 48 h. Cells were washed with 1 × PBS, centrifuged at 1500 rpm for 5 min and incubated with annexin V-FITC (Cat# V13241, Invitrogen) for 10 min. Cells were then stained with 10 μl of 40 μg/μl propidium iodide (PI) (Sigma, USA). The apoptosis level of cells was determined with a BD FACSCalibur flow cytometer (BD Biosciences, USA) and analyzed with Cell Quest software (BD Biosciences).

### Western blotting

Western blotting was performed as described previously [24]. Briefly, the total protein from lysates of U-87MG and U251 cells was extracted by re-suspending the cell pellets in RIPA buffer. Western blot analyses were performed with polyclonal antibodies against Pygo2, JNK, c-Jun and Bcl-2 (Santa Cruz Biotechnology, USA); monoclonal β-actin antibody was used as a control (Sigma, USA).

### Luciferase reporter gene assay

The transcription activity of AP-1 was tested using a PathDetect Signal Transduction Pathway AP-1 cis-Reporting system (Cat# 219073, Agilent Technologies, USA). Cells were transfected with 1 μg/μl pAP-1-Luc plasmid that contained the luciferase reporter gene driven by a basic promoter element plus a defined inducible cis-enhancer element. The transcriptional activity of ATF2 was assessed using a PathDetect Signal Transduction Pathway ATF2 trans-Reporting system (Cat# 219026, Agilent Technologies). Cells were transfected with 1 mg/ml pFA-ATF2 fusion plasmid fused with the yeast GAL4 DNA binding domain (DBD), residues 1-147. The fusion trans-activator plasmid contained the human cytomegalovirus promoter to drive constitutive expression of the trans-activator protein. Analyses were performed according to the manufacturer's protocol.

### Determination of caspases activity

The activity of caspase-3, −8 and −9 were measured using a Caspase-Glo^®^ 3/7 Assay (Cat# G8090, Promega, USA), Caspase-Glo^®^ 8 Assay (Cat# G8200, Promega, USA) and Caspase-Glo^®^ 9 Assay (Cat# G8210, Promega, USA). Cells were collected after treatment with PTX for 48 h, and then, reaction mixtures (100 mM HEPES, 10% sucrose, 0.1% CHAPS, 10 mM DTT, 1 mM EDTA, pH 7.4) containing appropriate dilutions of enzyme, proluminescent caspase-3 DEVD-aminoluciferin substrate (Ac-DEVD-AFC) or proluminogenic caspase-8 substrate (Ac-IETD-AFC) or proluminogenic caspase-9 substrate (Ac-LEHD-AFC) in a total volume of 100 μl were added to the cells. Related caspase activity was quantified by detection of the fluorescence of free AFC after cleavage from the peptide substrate, with Ex. = 400 nm and Em. = 505 nm, using a fluorometer. Analyses were performed according to the manufacturer's protocol.

### Measurement of rhodamine-123 efflux

P-gp activity in U-87MG and U251 cells was assessed based on rhodamine-123 (Rho-123) efflux. Cells were incubated with 0.5 μg/ml Rho-123 for 30 min at 37°C in DMEM w/o phenol-red, washed twice in ice-cold phosphate-buffered saline (PBS) and resuspended in medium. Samples were then incubated at 37°C for 15 min. Rho-123 fluorescence in cells was measured at 530 nm on a FACScan flow cytometer supplied with a 488 nm argon laser by measuring median fluorescence intensity (MFI).

### RNA extraction and reverse-transcription quantitative PCR (qRT-PCR)

RNA extraction and reverse-transcription quantitative PCR (qRT-PCR) were performed as described previously [24]. The following primers were used for MDR1 mRNA, 5′-GGAGGAGCAAAGAAGAAG-3′ and 5′-AATGTAAGCAGCAACCAG-3′; for Pygo2, 5′-AGAA AAGAAGCGAAGGAAGTCAAA-3′ and 5′-GGTGAT CCACCATGGGAGTT-3′; and for β-actin, 5′-CCAAGCA GCATGAAGATCAA-3′ and 5′-TCTGCTGGAAGGTG CTGAG-3′. β-actin was used as an internal control

### Cell migration and invasion assays

Cell migration and invasion assays were performed as described previously [52].

### MTT assay

MTT assays were performed as described previously [52].

### Chromatin immunoprecipitation assay

Chromatin immunoprecipitation assay (CHIP) was performed as described previously [24]. The primers used were 5′-GGAGCAGTCATCTGTGGTGA-3′ and 5′-CTCGAATGAGCTCAGGCTTC-3′.

### Statistical analysis

Statistical analysis was performed using SPSS 13.0 software for Windows. Differences in characteristics or values were examined by χ^2^ and Fisher's exact tests. Statistical significance was based on a *P*-value of < 0.05.
